# miRNA122a regulation of gene therapy vectors targeting hepatocellular cancer stem cells

**DOI:** 10.18632/oncotarget.25280

**Published:** 2018-05-04

**Authors:** Bijay Dhungel, Charmaine A. Ramlogan-Steel, Christopher J. Layton, Jason C. Steel

**Affiliations:** ^1^ Gallipoli Medical Research Institute, Greenslopes Private Hospital, Brisbane, QLD 4120, Australia; ^2^ Faculty of Medicine, The University of Queensland, Herston, Brisbane, QLD 4006, Australia; ^3^ University of Queensland Diamantina Institute, Translational Research Institute, Woolloongabba, QLD 4102, Australia

**Keywords:** hepatocellular carcinoma, cancer stem cells, targeted gene therapy, post-transcriptional targeting, miRNA122a

## Abstract

In this study, we report a miRNA122a based targeted gene therapy for hepatocellular cancer stem cells (CSCs). First, we assessed the levels of miRNA122a in normal human hepatocytes, a panel of hepatocellular carcinoma (HCC) cell lines and hepatocellular CSCs observing its significant downregulation in HCC and CSCs. The miRNA122a binding site was then incorporated at the 3’-UTR of reporter genes gaussia luciferase (GLuc) and eGFP which resulted in significant hepatocyte detargeting. Using this strategy for the delivery of gene directed enzyme prodrug therapy (GDEPT) utilizing the cytosine deaminase/5-fluorocytosine (CD/5-FC) system, we showed significant killing in cells with low or no miRNA122a while those cells, such as hepatocytes with high miRNA122a were largely spared. Next, we showed that CSC enriched tumorspheres exhibit a significant downregulation of miRNA122a expression providing a rational to exploit its binding site for targeted gene delivery. Using plasmids harboring reporters GLuc and eGFP with or without miR122a binding sites, we showed high reporter expression in the CSCs and little reported expression in the non-enriched cultures. Finally, we demonstrate the efficacy of miRNA122a based post-transcriptionally targeted GDEPT for hepatocellular CSCs.

## INTRODUCTION

Hepatocellular carcinoma (HCC) is the most prevalent form of primary malignancy in the liver and is the third leading cause of cancer related death worldwide [1, 2]. Currently, effective therapeutic treatment options for HCC are extremely limited. Liver transplantation and resection have the potential to be curative but are limited to early stage disease [3]. Less than 20% of HCC patients are diagnosed with early stage tumors [4]. Treatments for the vast majority of patients with unresectable tumors are largely palliative with recurrence often observed after therapeutic interventions [5].

Cancer stem cells (CSCs) represent a subpopulation of cancer cells with innate resistance to radio and chemotherapies and are vital for tumor progression and recurrence after therapy [6, 7]. Accumulating evidences suggest that a small subset of HCC possess these stem like properties and thus are important targets for therapeutic interventions [8]. Current strategies to target HCC derived CSCs include targeting cell surface markers like (e.g. CD133, CD44) [9], blocking pathways required for CSC survival (e.g. PI3K/AKT, Oct4) and self-renewal (Wnt, Notch, TGF-β) [8], targeting the tumor microenvironment [10], and induction of differentiation CSC differentiation [11] either with chemical compounds or nanoparticles [12]. In this study we use a targeted gene therapy approach to target the CSC population. Gene therapy has undergone a renaissance in the past few years boosted by the approval of gene therapy based drugs by both the European Union (EU) [13] as well as the Food and Drug Administration (FDA) [14]. Designing strategies to deliver therapeutic genes to the hepatocellular CSC population is a new and important approach for HCC therapy.

microRNAs (miRNAs) are small non-coding RNA molecules which post-transcriptionally regulate the expression of genes that are involved in a variety of cellular functions including development, maintenance and/or elimination of cancer stem cells by binding to its complementary site usually present at the 3′-UTR of the corresponding gene. A number of miRNAs have been reported to be dysregulated in HCC derived CSC [15]. miRNA122, accounting for about 70% of the total miRNA pool, has been reported to be significantly downregulated in HCC [16] and HCC derived CSCs [17]. Synthetic mimic miRNA and miRNA inhibitors are currently being tested in preclinical and clinical trials for various diseases including HCC [18, 19]. The use of miRNA as a therapeutic approach may, however, have inherent risks, the largest being its propensity to induce off-target silencing of non-related genes [20, 21]. As an alternative to the use of mimics and inhibitors to alter the expression of miRNA122a, the downregulation of miRNA122a in HCC and HCC derived CSC provides an opportunity for specific therapeutic gene delivery by incorporating its binding site at the 3’-UTR of a therapeutic gene [22].

In this study, we demonstrate the utility of a miRNA122a based strategy to target HCC and hepatocellular CSCs for gene delivery which is pivotal for targeted cancer gene therapy. We show that hepatocellular CSCs exhibit significant downregulation of miRNA122a and that including the miRNA122a binding site into the 3’-UTR of a vector results in significant hepatocyte detargeting and subsequent targeting of HCC and hepatocellular CSCs.

## RESULTS

### miRNA122a is downregulated in HCC and non-HCC cancer cell lines

**Figure 1:**
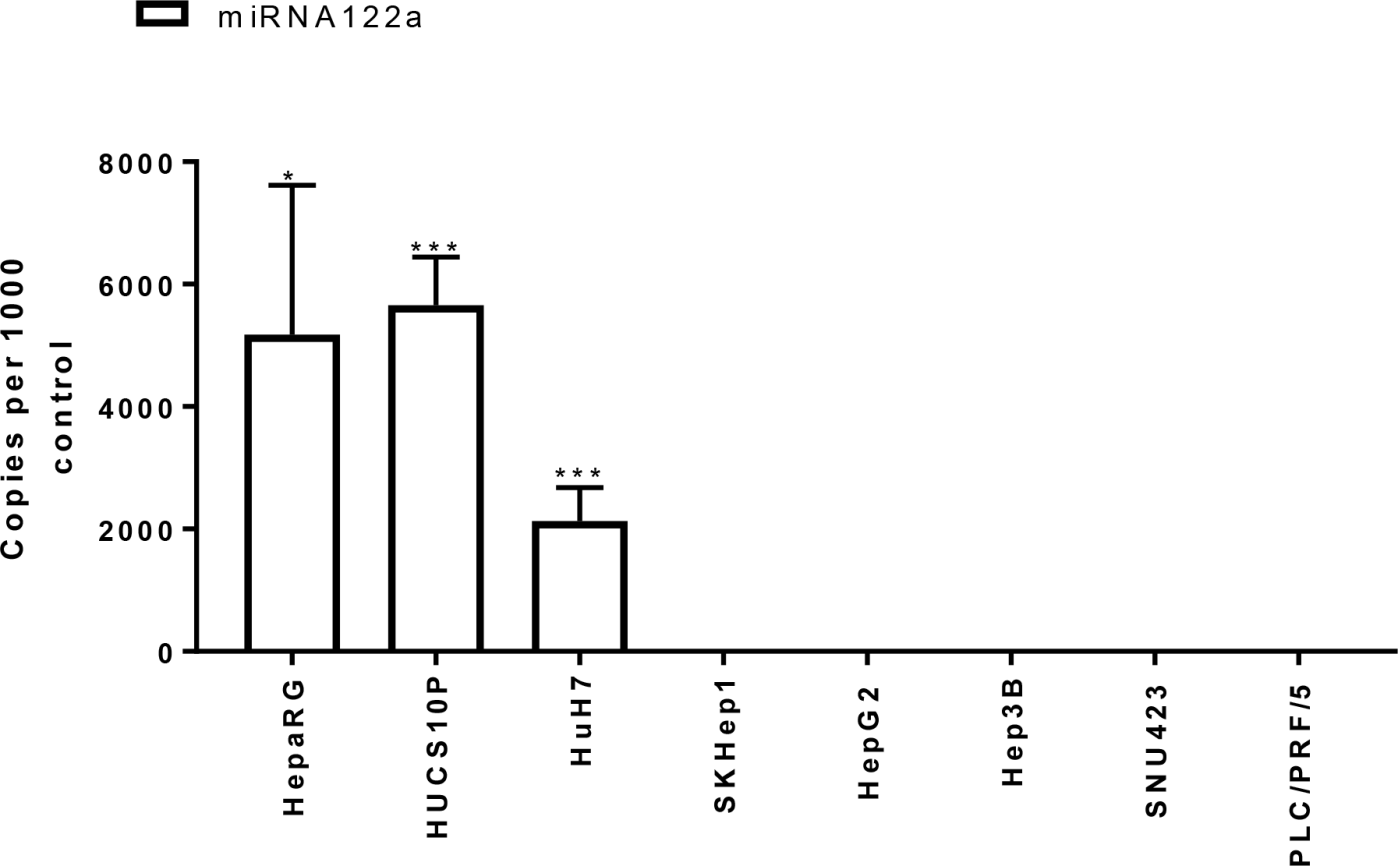
miRNA122a is downregulated in HCC and other cancer cell lines In order to quantify the expression levels of miRNA122a in hepatocytes and HCC cells, we isolated total RNA from primary human hepatocytes, HepaRG cells and HCC cell lines. qRT-PCR was then performed to determine the number of copies of miRNA122a which was reported as copy number per 1000 copies of control using the formula (2^(Ct control–Ct miRNA122a))*1000. Two-tailed *t*-test was performed between miRNA positive cells: HepaRG, HUCS10P, and HuH7 and miRNA negative HCC cells individually using Graph pad prism 7.0 (*n* > 3, ^*^ < 0.05, ^***^ < 0.005).

We quantified the levels of expression of miRNA122a in primary human hepatocytes, and a panel of HCC and non-HCC cell lines. The copy number of miRNA122a was reported as copies per 1000 copies of human positive control. Using this method, primary human hepatocytes were found to contain an average of 5655 copies of miRNA122a per 1000 copies of control while the expression of miRNA122a was not detected in HCC cell lines Hep3B, SKHep1, PLC/PRF/5, and SNU423 (Figure 1). Similarly, the expression of miRNA122a was undetected in all the non-HCC cell lines examined (data not shown). However, HCC cell line HuH7 was found to be miRNA122a positive with 2130 copies of miRNA122a per 1000 copies of control (Figure 1). These results confirmed the hepatocyte-specific nature of miRNA122a and established its downregulation in HCC cell lines providing a rationale for exploiting the binding site for targeted gene delivery.

### Inclusion of miRNA122a binding sites at the 3′-UTR of a transgene enables efficient hepatocyte detargeting

**Figure 2:**
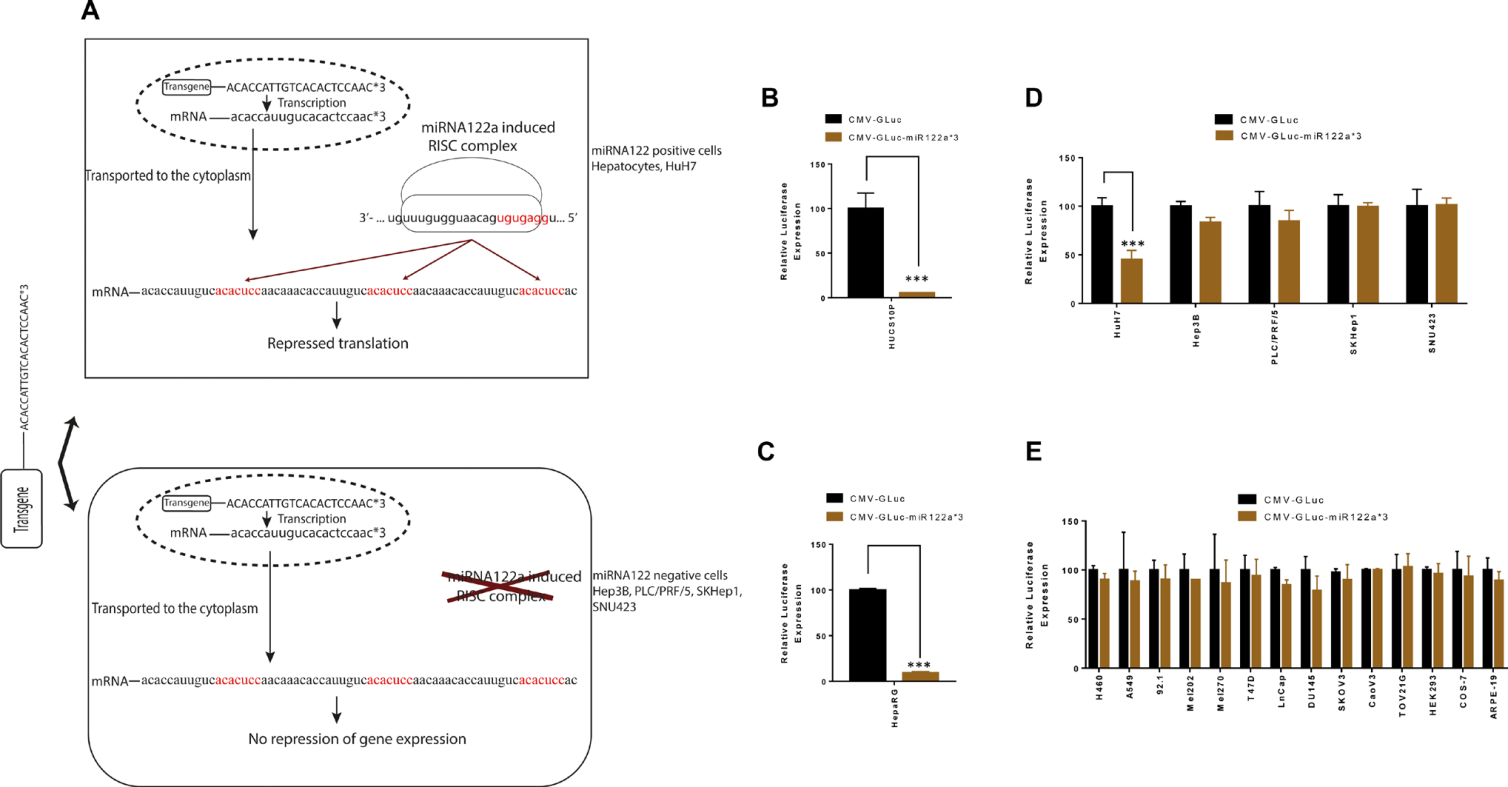
Inclusion of miRNA122a binding sites at the 3′-UTR of a transgene can inhibit its expression in hepatocytes To study the effectiveness of incorporating miRNA122a binding sites at the 3′-UTR for hepatocyte detargeting, we constructed expression plasmids harboring GLuc reporter driven by the ubiquitous CMV promoter (CMV-GLuc) as well as plasmids harboring CMV driven reporter with 3 miRNA122a binding sites at the 3′-UTR (CMV-GLuc-miR122a*3). These plasmids were transfected into human hepatocytes, HCC, and non-HCC cell lines. (**A**) Shows the principles of miRNA based detargeting: In cells with endogenous miRNA122, the mRNA with miRNA122 binding sites is transported to the cytoplasm where it interacts with miRNA122 induced RISC complexes by sequence complementary binding to the seed sequence (in red). This mediates gene suppression through mRNA degradation and/or translational repression. In cells that do not have endogenous miRNA122a (such as HCC), there is no binding to the miRNA sites on the transgene and translation occurs uninhibited. (**B**) Primary human hepatocytes were transfected with CMV-GLuc and CMV-GLuc-miR122a*3 and the amount of luciferase secretion was quantified. Luciferase expression for CMV-GLuc-miR122a*3 was reported as a percentage of CMV-GLuc for each cell type. These transfection experiments were also performed for (**C**) HepaRG, (**D**) HCC and (**E**) non-HCC cell lines. The difference between CMV-GLuc and CMV-GLuc-miR122a*3 was compared with two-tailed *t*-test for statistical significance using Graph Pad Prism. (*n* > 3, ^***^, *p* < 0.001).

We constructed expression plasmids harboring reporter genes GLuc and eGFP with 3 miRNA122a binding sites at the 3′-UTR. These plasmids were transfected in miRNA122a positive primary hepatocytes and HuH7 cell line as well as miRNA122a negative HCC and non-HCC cell lines. It was predicted that, in the miRNA122a positive cells, the reporter mRNA with miRNA122 binding sites would be transported to the cytoplasm where it can interact with endogenous miRNA122 induced RISC complexes by sequence complementary binding resulting in translational repression (Figure 2A). In cells that do not have endogenous miRNA122a (such as HCC), there is predicted to be no binding to the miRNA sites on the transgene and translation would occur uninhibited.

When we examined this experimentally, the relative luciferase expression (normalized to CMV-GLuc) was significantly inhibited in primary human hepatocytes after transfection with CMV-GLuc-miR122a*3 (*p* < 0.001) (Figure 2B, 2C). Similarly, a 55% reduction in luciferase expression was observed in HuH7 cell line (*p* < 0.001) (Figure 2D). In contrast, the miRNA122a negative HCC and non-HCC cell lines displayed no significant differences in luciferase expression (Figure 2D and 2E).

### Inhibition of miRNA122a in hepatocytes rescues the expression of transgene containing its binding site at the 3′-UTR while its overexpression in HCC cells lead to the suppression of transgene expression

**Figure 3:**
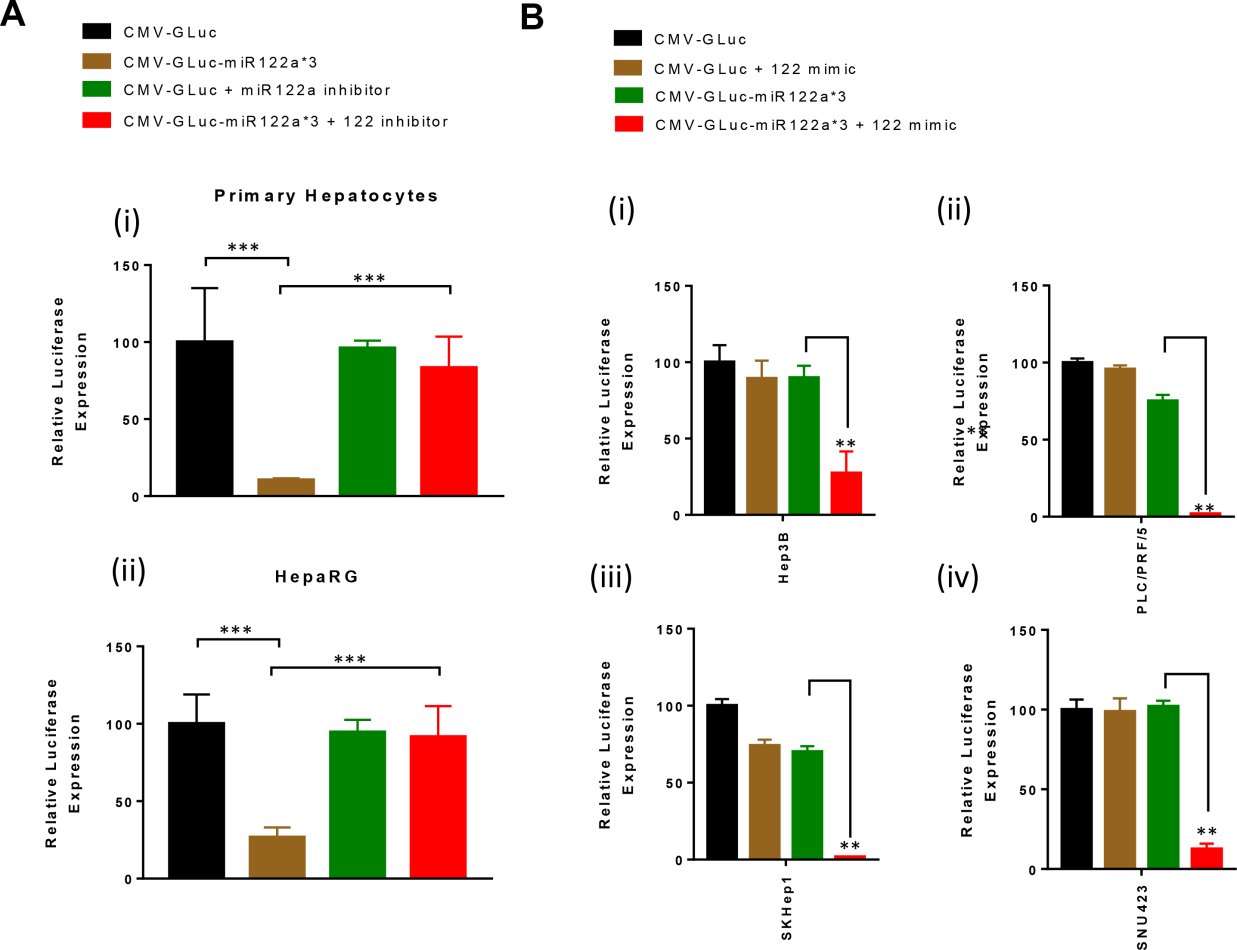
Inhibition of miRNA122a in hepatocytes rescues the expression of transgene with miRNA122a binding site at the 3′-UTR, whereas overexpression of miRNA122a in HCC cell lines inhibits it (**A**) In order to study the effect of inhibition of miRNA122a in hepatocytes on the expression of transgene with miRNA122a binding sites, we co-transfected human hepatocytes (i) and HepaRG (ii) with CMV-GLuc-miR122a*3 and miRNA122a inhibitor, and CMV-GLuc and miR122a inhibitor and quantified the amount of luciferase secreted. The expression of luciferase was quantified for each group and expressed as a percentage of CMV-GLuc. (**B**) Similarly, HCC cell lines Hep3B, PLC/PRF/5, SKHep1, and SNU423 were co-transfected with CMV-GLuc-miR122a*3 and miRNA122a mimic, and CMV-GLuc and miRNA122a mimic; secreted GLuc was quantified for each group and reported as a percentage of CMV-GLuc. Two-tailed *t*-test was performed to determine the statistical significance of the observed differences between groups. (*n* > 3, ^***^p < 0.001, ^**^p < 0.005).

In order to confirm that miRNA122a expression was responsible for the detargeting of our vectors, we utilized miRNA122a inhibitors and mimics to alter miRNA expression in hepatocyte and HCC cell lines. Using the miRNA122a inhibitor, we showed a significant increase in luciferase expression in both primary human hepatocytes (Figure 3A (i)) and HepaRG (Figure 3A (ii)) following transfection with CMV-GLuc-miR122a*3 (*p* < 0.001). The addition of miRNA122a inhibitors did not alter the luciferase expression in the low/non-expressing HCC cell lines (data not shown).

Using a miRNA122a mimic to increase the presence of miRNA in the HCC cell lines, we showed a significant decrease in luciferase expression following transfection with CMV-GLuc-122a*3 (*p* < 0.005). Luciferase expression decreased from 89.8% to 27.3%, 75% to 1.8%, 70% to 1.7%, and 101% to 12% (*p* < 0.005) in HCC cell lines Hep3B, PLC/PRF/5, SKHep1 and SNU423 respectively (Figure 3B). The addition of miRNA122a mimic did not alter the luciferase expression in the primary human hepatocytes (data not shown). Taken together, these results confirmed that the observed decrease in reporter expression in miRNA122a expressing cells after transfection with CMV-GLuc-miR122a*3 was due to the action of miRNA122a.

### miRNA122a regulated gene directed enzyme prodrug therapy (GDEPT) based on cytosine deaminase/5-fluorocytosine (CD/5-FC) can induce HCC-specific cell death

**Figure 4:**
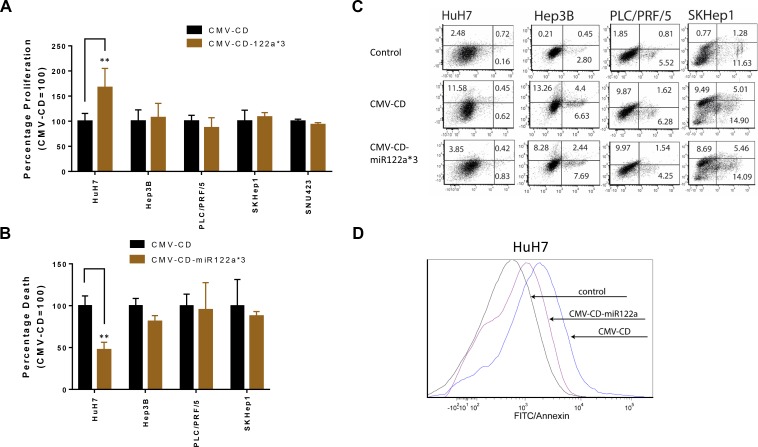
miRNA122a based post-transcriptionally controlled suicide gene therapy for HCC To explore the possibility of performing a targeted gene directed enzyme prodrug therapy (GDEPT) utilizing cytosine deaminase/5-Fluorocytosine (CD/5-FC) system; we constructed CMV-CD, and CMV-CD-miR122a*3; the later consisting of 3 miRNA122a binding sites at the 3′-UTR of suicide gene CD. miRNA122a positive cell line HuH7 and miRNA122a negative HCC cell lines were transfected with these plasmids and subsequently incubated with the prodrug 5-FC. (**A**) The percentage proliferation was quantified with MTS assay and proliferation percentage of cells transfected with CMV-CD-miR122a*3 was reported as percentage of those transfected with CMV-CD. (**B**) Similarly, total cell death after suicide gene therapy with CMV-CD-miR122a*3 was quantified with flow cytometry by annexin/PI co-staining and reported as relative to CMV-CD. (**C**) Representative flow cytometric plots for each cell type showing percentage of annexin and PI positive cells after suicide gene therapy (**D**) Representative flow cytometric plot of HuH7 cell line showing annexin positive cells in the control group overlayed with annexin positive cells after transfection with CMV-CD and CMV-CD-miR122a*3. Two-tailed *t*-test was performed in Graph Pad prism to check for statistical significance of observed differences. (*n* > 3, ^**^p < 0.005).

In order to study the feasibility of targeted GDEPT based on differential miRNA122a expression, we constructed vectors with the suicide gene cytosine deaminase (CD) and examined their ability to inhibit cellular proliferation and induce cytotoxicity. CD functions by converting the prodrug 5-FC into a cytotoxic metabolite. In the proliferation study, cells transfected with CMV-CD-miR122a*3 and incubated with 5-FC were normalized against proliferation observed with cells transfected with an untargeted CMV-CD construct. The HuH7 cell line, expressing miRNA122a at similar levels to that of normal hepatocytes, transfected with CMV-CD-miR122a*3 had a significantly higher proliferation than those transfected with the untargeted CMV-CD constructs (*p* < 0.005) whereas the miRNA122a negative HCC cell lines showed no significant difference in proliferation (Figure 4A).

Next, we performed flow cytometric quantification of cell death based on annexin/PI staining after transfection with CMV-CD-miR122a*3. The relative cell death of HuH7 after transfection with CMV-CD-miR122a*3 and incubation with 5-FC was significantly inhibited in HuH7 cell line (*p* < 0.005), whereas no significant difference in cell death was observed between groups CMV-CD and CMV-CD-miR122a*3 in HCC cell lines Hep3B, PLC/PRF/5, SKHep1 (Figure 4B–4D). Taken together these results show that HCC with limited miRNA122a expression can be therapeutically targeted with CMV-CD-miR122a*3 constructs.

### miRNA122a is downregulated in cancer stem cells enriched HCC tumorspheres

**Figure 5:**
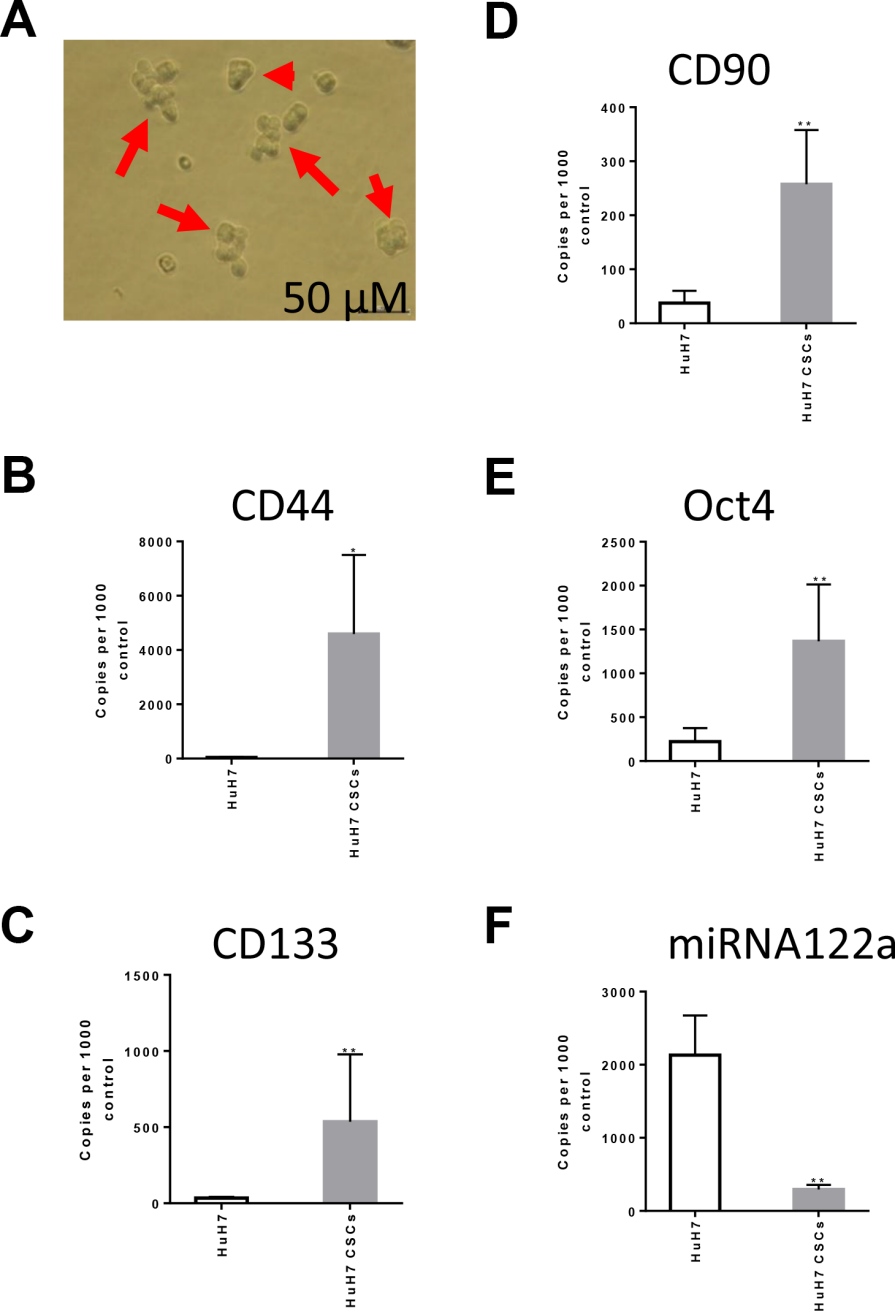
miRNA122a is significantly downregulated in HCC stem cell enriched tumorspheres To study the expression profile of miRNA122a in stem cells derived from miRNA122a positive HuH7 cell line, we maintained the cells in a stem cell enriching NSA media as 3D tumorspheres. (**A**) Phase contrast images of tumorspheres captured at day 5 after seeding HuH7 in NSA media. The cells were collected and total cDNA was synthesized from total cellular RNA. qRT-PCR was then performed to quantify the levels of stemness related genes (**B**) CD44, (**C**)CD133, and (**D**) CD90 (**E**) Oct4 which were reported as copy number per 10,000 copies of GAPDH house keeper using the formula (2^(Ct housekeeper- Ct sample))*10000. (**F**) The expression levels of miRNA122a was expressed as copy number per 10,000 copies of control. Difference between two groups was tested with a two-tailed *t*-test using Graph Pad Prism. (*n* > 3, ^*^, *p* < 0.05, ^**^, *p* < 0.005).

We then investigated the miRNA122a expression profile in HuH7 cultures enriched for hepatocellular CSCs. We cultured 3D tumorspheres of HuH7 in a serum-free NSA media (Figure 5A) and quantified the expression levels of HCC stemness markers CD44 (Figure 5B), CD133 (Figure 5C), CD90 (Figure 5D), and Oct4 (Figure 5E), as well as miRNA122a (Figure 5F). When compared to the adherent HuH7 cells, the CSC enriched tumorspheres of HuH7 showed a significant increase in the expression of hepatocellular CSC markers CD44 (*p* < 0.05), CD133 (*p* < 0.005), CD90 (*p* < 0.005), and Oct4 (*p* < 0.005) (Figure 5B–5E) normalized to GAPDH housekeeper. Concurrent with the increase in stemness markers, we observed a 4.34 fold decrease in expression of miRNA122a (*p* < 0.005) in HuH7 cultures enriched for CSCs (Figure 5F). These results suggested that under the culture conditions used, an enrichment of cancer stem cells is achieved which can be used as a model for studying characteristics of HCC CSCs. Furthermore, an inhibition of miRNA122a was observed in these HCC CSCs indicating a downregulation of miRNA expression in CSCs derived from HCC subpopulation without miRNA122a inhibition providing a rational for miRNA122a binding site based targeting of these particular CSCs.

### Incorporation of miRNA122a binding site in at the 3’-UTR of the expression cassette can lead to efficient targeting of HCC cancer stem cells

**Figure 6:**
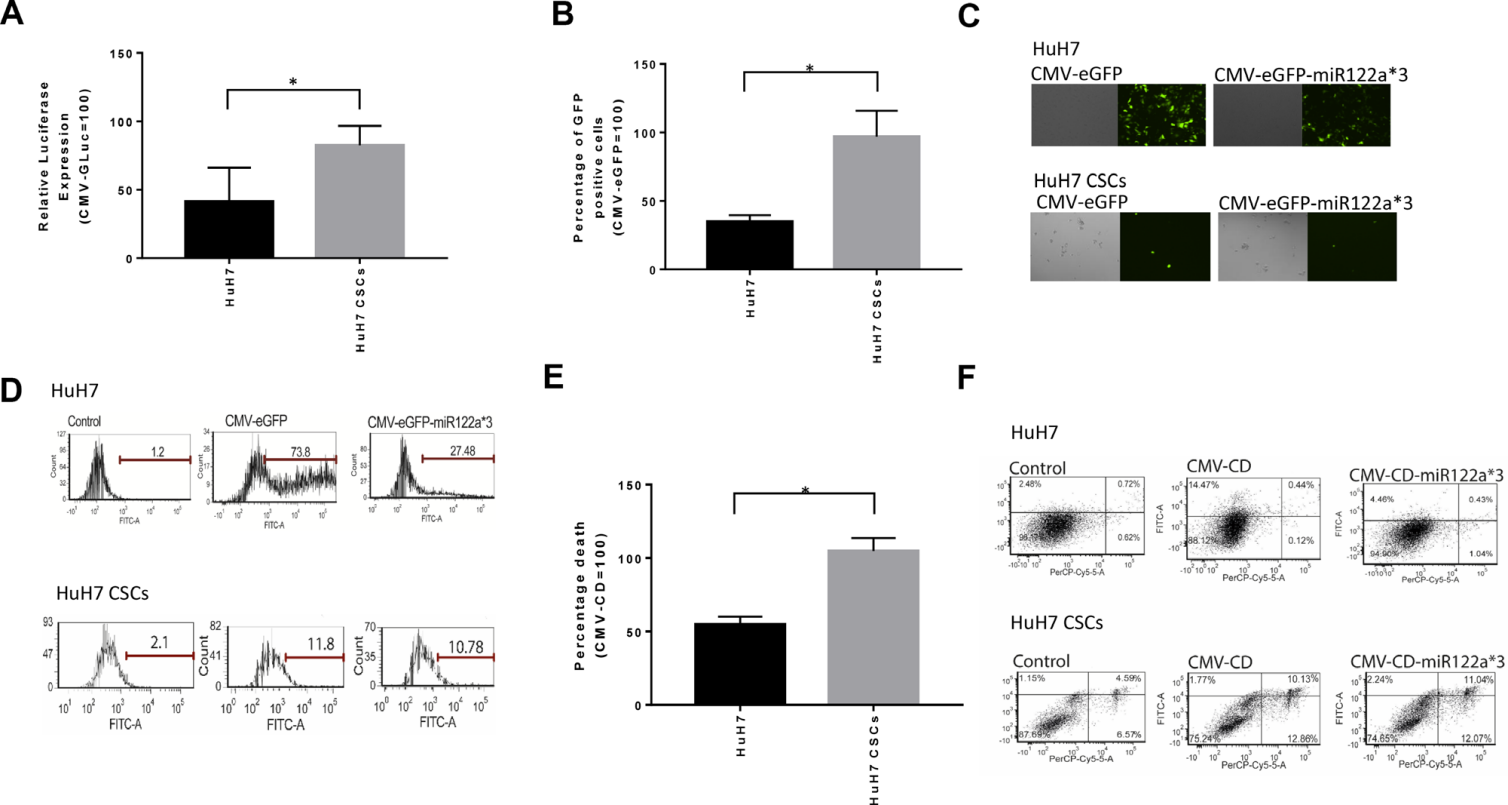
Downregulation of miRNA122a in stem cell enriched tumorspheres of HuH7 allows efficient targeting of these stem cell population After observing a downregulation of miRNA122a in stem cell enriched tumorspheres of HuH7, we transfected the tumorspheres with (**A**) CMV-GLuc, and CMV-GLuc-miR122a*3 and (**B**) CMV-eGFP, and CMV-eGFP-miR122a*3. The amount of secreted luciferase after transfection with CMV-GLuc-miR122a*3 was reported as a percentage of CMV-GLuc and the percentage of GFP positive cells after transfection with CMV-eGFP-miR122a*3 was reported as relative to CMV-eGFP both for adherent HuH7 as well as HuH7 tumorspheres. (**C**) Representative fluorescent microscope images of HuH7 and HuH7 CSCs transfected with CMV-eGFP and CMv-eGFP-miR122a*3 (**D**) Representative flow cytometry images showing GFP-positive images after transfection of HuH7 and HuH7 CSCs with CMV-eGFP and CMv-eGFP-miR122a*3 (**E**) HuH7 and HuH7 CSCs were transfected with CMV-CD, and CMV-CD-miR122a*3 and incubated with 5-FC containing media. Total cell death was then quantified with annexin/PI co-staining. The observed percentage total apoptotic cells after suicide gene therapy with CMV-CD-miR122a*3 was reported as a percentage of that with CMV-CD both for HuH7 and HuH7 CSCs. (**F**) Representative flow cytometric images of HuH7, and HuH7 CSCs after suicide gene therapy showing annexin and PI positive cells. For statistical analysis, two-tailed *t*-test was performed to compare the observed differences between HuH7 and HuH7 CSCs. (*n* > 3, ^*^, *p* < 0.05).

Next, we investigated whether the miRNA122a binding site could be utilized for targeting CSCs derived from HCC. To do this, we transfected HuH7 and HuH7 CSCs with CMV-eGFP-miR122a*3, and CMV-GLuc-miR122a*3 and normalized the expression of reporters with CMV-eGFP and CMV-GLuc respectively. We showed that HuH7 cultures, enriched for CSCs, had a significant increase in the expression of GLuc (Figure 6A) and eGFP (Figure 6B–6D) with 2-fold higher GLuc expression and 2.78-fold higher GFP expression (*P* < 0.05).

Finally, we examined whether HuH7 CSCs could be targeted and killed using CMV-CD-miR122a*3 constructs. We showed that HuH7 tumorspheres, when transfected with CMV-CD-miR122a*3 and incubated with 5-FC, had significantly more cell death than those cultures not enriched for CSCs (*P* < 0.05) with a 1.91 fold higher cell death observed (Figures 6E–6F). Collectively, these results suggested an efficient miRNA122a based post-transcriptionally targeted gene therapeutic approach for hepatocellular CSCs.

## DISCUSSION

Rationally designed targeted gene therapy is an attractive therapeutic approach for diseases like HCC which requires a tumor specific action in order to prevent significant off-target effects in an already compromised liver. One of the different strategies for targeted gene delivery is the modification of the therapeutic payload (therapeutic gene cassette) by including disease specific regulatory elements like tumor specific promoters and miRNA binding sites [22, 23]. In recent years, there has been accumulating evidence demonstrating the efficacy of utilizing binding sites of miRNAs downregulated in cancers as an effective approach [24–27]. Given that miRNAs play a pivotal role in virtually every cellular pathway, it is conceivable that they are involved in the development and maintenance of cancer stem cells, thought to be responsible for resistance to chemo and radiotherapies as well as tumor recurrence and metastasis. Indeed, a number of miRNAs including miR18a, miR155, miR130b, miR125, miR216a/217 have roles in maintaining HCC stemness by targeting genes like CDX2, GATA6, NLK and controlling key pathways required for stemness [8, 28].

The well-known intra and inter-tumoral heterogeneity of HCC means that not all subtypes may have a reduced miRNA122a profile [29, 30]. When screening our panel of HCC cell lines, we found that the reduction of miRNA122a, while occurring in most lines, did not occur in all cell lines with HuH7 being the outlier. Interestingly, however, we showed that HuH7 cultures enriched for CSCs had significantly reduced miRNA122a expression. The link between miRNA122a expression and CSC has been shown in both transgenic mice and cell lines. Transgenic mice with miRNA122a knockout have been shown to spontaneously develop HCC with the miRNA122a −/− tumor cells showing increased expression of CSC markers such as EpCAM, CD90 and CD133 [31]. CD133 has been shown to be a direct target gene of miRNA122a and its knockdown can supress stem-like characteristics, suggesting that miRNA122a may have a role in hepatocellular CSC self-renewal and proliferation [31]. In a recent report by Song *et al.* it was shown that CD133^+^ CSCs isolated from the human HCC cell line, PLC/PRF/5 also exhibited a significant reduction in miRNA122a compared to non-CSCs [17]. In this study they showed that in hepatocellular CSCs, miRNA122a plays a key role in negatively regulating glycolysis through its interaction with PDK4 and that the return of miRNA122a to the CSC population inhibits their stemness [17]. Given the importance of miRNA122a in the maintenance, development and initiation of HCC, its liver specificity, and differences in expression levels of miRNA122a in HCC and CSCs, we concluded that there is a strong rationale to explore the efficacy of miRNA122a for post-transcriptional targeting of HCC.

Exploiting the differential expression of miRNA122a between functional hepatocytes, HCC and hepatocellular CSCs, we designed vectors incorporating 3 copies of the binding site of miRNA122a with the goal of limiting expression of transgenes to those cells with reduced miRNA122a expression, i.e. HCC and hepatocellular CSCs. Using luciferase as a marker transgene, we showed that cells expressing miRNA122a (primary hepatocytes and HuH7) had a significant decrease in luciferase expression. Conversely, HCC cells with little or no miRNA122a had significantly higher luciferase expression. These results were in line with previous studies exploiting the miRNA122a binding site for efficient hepatocyte detargeting [32, 33]. After establishing the efficacy of miRNA122a binding site based targeting of HCC, we examined whether a similar targeting approach could be used to direct transgene expression to hepatocellular CSCs. To do this we utilized 3D tumorspheres cultures of HuH7 to enrich for CSCs [34] and showed increases in stemness markers and a concurrent downregulation of miRNA122a. The CSC enriched cultures were readily transfected with luciferase and eGFP vectors with or with the miRNA122a binding sites and showed enhanced transgene expression in the CSCs compared to the non-enriched, miRNA122a expressing cultures. These results confirmed that miRNA122a targeting of hepatocellular CSC is possible. While similar approaches have been used for targeting cancer including breast [35], glioblastoma [36, 37], fibrosarcoma [38] and HCC [32, 33], this is the first use of this type of approach to target CSCs.

To explore the potential therapeutic benefit of using miRNA122a binding sites in HCC, we constructed vectors expressing the suicide gene cytosine deaminase (CD) with or without the miRNA binding sites (pCMV-CD-miR122a*3 and pCMV-CD). The CD gene produces an enzyme that converts the non-toxic 5-FC prodrug to the cytotoxic 5- fluorouracil (5-FU). We found that the use of post-transcriptional targeting of HCC and the subsequent detargeting of hepatocytes using miRNA122a was both feasible and effective, with significantly more killing and the inhibition of proliferation in HCC cells. Cell death and inhibition of proliferation was directly related to the presence or absence of miRNA122a in the cells. Using the pCMV-CD-miR122a*3 vectors in the CSC enriched cultures, we also showed significant cytotoxicity in line with miRNA122a expression and confirmed its utility in targeting CSCs. By targeting CSCs with a suicide gene therapy approach, such as the CD/5-FC system used in this model, there is the potential for a double bystander effect. The first induced by the CD/5-FC itself, where the non-toxic 5-FC is processed by the CSCs expressing CD into the toxic anabolite 5-fluorouracil (5-FU) [39]. 5-FU then freely diffuses across the cellular membrane and spreads to adjacent tumor cells that do not express the CD gene [39, 40]. The second bystander effect has the potential to remove both the bulk tumor and the CSCs [40]. Given that CSCs drive tumor progression and maintenance, their removal can result in tumor degeneration and possibly reduced recurrence [9]. The potential benefits of bystander effects inherent with the CD/5-FC system was seen in our 3D sphere cultures, representing a solid tumor mass, where increased tumor killing was evident.

The increasing knowledge of the genetic makeup of HCC and the CSCs derived from them allows identification of specific expression patterns which make them different from the normal liver cells. Initial work on utilising this knowledge for targeted gene therapy centred on the use of HCC restricted tumour specific promoters, such as that for alpha- fetoprotein (AFP), to drive transgene expression [41]. The use of this type of transcriptional targeting has, however, failed to progress to clinical trials. The low transcriptional activity, particularly when compared to promoters such as CMV/CAG [42] and problems with their specificity [23, 43] when used alone, has played a role in this failure. Post-transcriptional targeting through the use of miRNA avoids the problems associated with the low transcriptional activity as any promoter can be used and specificity can be potentially increased through the use of TS of multiple miRNAs. This makes miRNA targeting potentially more promising in terms of moving this technology into the clinic. In many HCC and hepatocellular CSCs, miRNA122a is down-regulated compared to normal or diseased liver hepatocytes. This proof-of-principle study shows that exploiting miRNA122a allows a tumour or CSC-specific delivery of transgene, thus limiting dose related toxicities to normal liver. It should be noted that miRNA122a is liver specific therefore additional liver targeting strategies such as the use of modified adeno associated viruses [23] will be necessary if used systemically.

## MATERIALS AND METHODS

### Cell culture

miRNA122a negative HCC cell lines Hep3B, PLC/PRF/5, SKHep1, and SNU423 were obtained from ATCC and maintained in DMEM (Thermo Fisher Scientific, Scoresby, Australia) media supplemented with 10% fetal bovine serum (FBS) (Gibco, Australia) and 1% penicillin/streptomycin (P/S) (Gibco). miRNA122a positive HCC cell line HuH7 was provided by Dr. Kim Bridle (University of Queensland). Cell line ID service was provided by the Australian Genome Research Facility (AGRF) and cell line genotyping was performed to confirm identity of cell lines. Breast cancer cell line T47D and prostate cancer cell lines LNCaP and DU145 were maintained in DMEM media with 10% FCS and 1% P/S. Melanoma cell lines 92.1, Mel202, Mel270 (gifted by Nicholas Hayward) and ovarian cancer lines SKOV3, CAOV3, TOV21G were cultured in RPMI media (Thermo Fisher Scientific) supplemented with 10% FCS and 1% P/S. Human retinal pigmental epithelium cell line ARPE-19 was maintained in DMEM/F12 media (Thermo Fisher Scientific) with 10% FCS and 1% P/S. Cryopreserved human hepatocytes (HUCS10P) (Lonza, Australia) containing pooled hepatocytes from 10 donors were cultured as per the manufacturer's recommendations in human hepatocyte maintenance media.

### Tumorsphere culture

3D tumorspheres of HuH7 were maintained in a serum free, stem cell conditioned NSA media as previously described [44]. The NSA media consisted of DMEM/F12 supplemented with 20 ng/ml recombinant human epidermal growth factor (rhEGF) (Lonza), 10 ng/ml recombinant human basic fibroblast growth factor (rhFGF) (Lonza), 1% P/S (Thermo Fisher Scientific), bovine serum albumin (BSA) (Sigma Aldrich, St. Louis, MO, USA), and 4 μg/ml heparin sulfate (Sigma Aldrich). Briefly, 30,000 cells were collected, washed thrice with PBS and seeded in the 24 well ultra-low attachment plate (Corning, NY, USA). Images of the tumorspheres were taken with a digital camera (Olympus DP21, Japan) connected to an inverted microscope (Olympus CKX41) using imaging software (CellSens, Olympus, Japan).

### Quantification of miRNA levels

Endogenous expression levels of miRNA122a was quantified by quantitative real time PCR (qRT-PCR) with Bioline Lo-Rox Sybr (Bioline, Alexandria, Australia) using the ViiA7 RT-PCR machine (Thermo Fisher Scientific) at following conditions: 95° C- 2mins followed by 40 cycles of 95° C- 5 s, 60° C -15 s and 70° C -15 s on the cDNA which was synthesized from total RNA isolated with trizol using the MystiCq microRNA cDNA Synthesis Mix (Sigma Aldrich) as per the manufacturer's recommendations. MystiCq Universal PCR (MIRUP, Sigma) and 5′- TGGAGTGTGACAATGGTGTTTGT- 3′ primers were used and the levels if miRNA122a was calculated as number of copies per 1000 copies of human positive control using formula (2^(Ct control-Ct sample))*1000. Similarly, HCC stemness markers CD44, EpCAM, and Oct4 were quantified as number of copies per 1000 copies of GAPDH housekeeping gene using primers listed in Table 1.

**Table 1:** Primers used for qRT-PCR studies

Gene	Forward primer	Reverse Primer
GAPDH	5′-TCCTGCACCACCAACTGCTTAGC-3′	5′- GCCTGCTTCACCACCTTCTTGAT-3′
CD44	5′-CCAGAAGGAACAGTGGTTTGGC-3′	5′-ACTGTCCTCTGGGCTTGGTGTT-3′
CD90	5′-GAAGGTCCTCTACTTATCCGCC-3′	5′-TGATGCCCTCACACTTGACCAG-3′
CD133	5′-CACTACCAAGGACAAGGCGTTC-3′	5′-CAACGCCTCTTTGGTCTCCTTG-3′
Oct4	5′-CTTCTGCTTCAGGAGCTTGG-3′	5′-GAAGGAGAAGCTGGAGCAAA-3′

### Construction of expression plasmids

The gene encoding gaussia luciferase (GLuc) with three miRNA122a binding sites (ACACCATTGTCACACTCCAAC*3) at the 3′-UTR was artificially synthesized (Thermo Fisher Scientific). The gene with and without miRNA binding sites was then cloned in the pscAAV-GFP (a gift from John T Gray, Addgene plasmid # 32396) using enzymes EcoRI, StuI and EcoRI, EcoRV to obtain CMV-GLuc and CMV-GLuc-miR122a*3 (Supplementary Information) respectively. Similarly, artificially synthesized cytosine deaminase (CD) was cloned in the above mentioned plasmids replacing GLuc to obtain CMV-CD and CMV-CD-miR122a*3.

### Transfection and reporter assays

All transfection studies for studying GLuc and eGFP expression were performed with Lipofectamine 3000 (Thermo Fisher Scientific) as per the manufacturer's protocol. Briefly, 30,000 cells were seeded in a 24 well plate and transfection was performed with 500 ng of plasmids. 72 hours post-transfection, the amount of secreted GLuc was quantified with the Pierce gaussia luciferase glow assay kit (Thermo Fisher Scientific) as per the manufacturer's recommendations. The chemiluminescence measurement was done with the Infinite 200 Pro NanoQuant (Tecan Trading AG, Switzerland). For primary human hepatocytes, the transfections were performed an hour after seeding and the amount of secreted GLuc was measured after 24 hours. In order to account for the difference in transfection efficiencies across cell lines, chemiluminescence detected with CMV-GLuc-miR122a*3 was normalized against that with CMV-GLuc for individual cell type. Tumorspheres of HuH7 were transfected at day 3 after seeding in the NSA media and secreted GLuc was quantified after 48 hours. Similarly, to quantify the percentage of eGFP positive cells, flow cytometry was performed with the FACS Canto II and data was analysed with FCS express version 3 (BD Biosciences; North Ryda, Australia). Percentage of GFP positive cells after transfection with CMV-eGFP-miR122a*3 was normalized with CMV-eGFP.

### Inhibition and overexpression of miR122a

miRNA122a inhibition experiments in primary hepatocytes were performed by co-transfecting miRNA122a inhibitor (Life technologies, Mulgrave Australia) (5pmol) and either CMV-GLuc or CMV-GLuc-miR122a*3 in a 24 well plate using Lipofectamine 3000 as per the manufacturer's protocol. 24 hours post transfection, secreted GLuc was quantified with Pierce gaussia luciferase glow assay kit. Similarly, HCC cell lines Hep3B, SKHep1, PLC/PRF/5, and SNU423 were co-transfected with miRNA122a mimic (4464066, Life Technologies) (5 pmol) and either CMV-GLuc or CMV-GLuc-miR122*3. The amount of secreted GLuc for each group was reported as a percentage of CMV-GLuc.

### Cell proliferation assay

For cell proliferation assays, 10,000 cells were transfected with CMV-CD or CMV-CD-122a*3 in a 96 well plate and media containing 10 μm 5-FC was added after 24 hours. CellTiter96 Aqueous One Solution Cell Proliferation Assay kit (Promega Corporation, Madison, WI USA) was used to quantify cell proliferation after 48 hours of incubation with 5-FC containing media. Absorbance was measured at 540 nm with the Infinite 200 Pro NanoQuant. Percentage proliferation was calculated for CMV-CD and CMV-CD-122a*3 for each cell line and percentage proliferation with CMV-CD-122a*3 was reported relative to CMV-CD in order to account for the difference in transfection efficiencies across cell lines.

### Cell death assay with Annexin V/PI

To quantify cell apoptosis, flow cytometry based on Annexin/PI staining was used (Life Technologies). 30,000 cells were transfected with CMV-CD and CMV-CD-122a*3 in a 24 well plate and incubated for either 24 hours (for tumorspheres) or 48 hours (adherent cells) with 10 μm 5-FC containing media. Cells were then washed with PBS, collected and co-stained with Annexin V and PI as recommended by the manufacturer. Annexin V/PI positive cells were quantified with the FACS Canto II and data analysis was performed with FCS express 3. The percentage of apoptotic cells after transfection with CMV-CD-122a*3 was normalized with CMV-CD.

### Statistical analysis

All experiments were repeated at least thrice in triplicates and results were presented as mean ± SD. Two-tailed student's *t*-test was performed to test the difference between groups (GraphPad Prism v7) (^*^ < 0.05, ^**^ < 0.01, ^***^ < 0.001).

## SUPPLEMENTARY MATERIALS FIGURES AND TABLES



## Abbreviations

CD: Cytosine deaminase
CSC: cancer stem cells
FC: fluorocytosine
GDEPT: gene directed enzyme prodrug therapy
HCC: hepatocellular carcinoma
GLuc: gaussia luciferase
miRNA: microRNA
UTR: untranslated region
