# Supplementary material for: miRNA122a regulation of gene therapy vectors targeting hepatocellular cancer stem cells
Source: Oncotarget. 2018 May 4;9(34):23577–88. doi: 10.18632/oncotarget.25280 (PMC5955118; doi:10.18632/oncotarget.25280)
Supplement: Supplementary file 1 [file oncotarget-09-23577-s001.pdf]

miRNA122a regulation of gene therapy vectors targeting hepatocellular cancer stem cells

SUPPLEMENTARY MATERIALS

Supplementary Information

Sequence of the plasmid construct (pscCVM-GLuc-miR122a*3)

Gluc coding region, flanking sequence with restriction sites (underlined), and miRNA122a binding sites (bold)

ATGGGAGTCAAAGTTCTGTTTGCCCTGATCTGCATCGCTGTGGCCGAGGCCAAGCCACCGAGAACAACG
AAGACTTCAACATCGTGGCCGTGGCCAGCAACTTCGCGACCACGGATCTCGATGCTGACCGCGGGAGTTGCCC
GGCAAGAAGCTGCCGCTGGAGGTGCTCAAAGAGATGGAAGCCAATGCCCGGAAAGCTGGCTGCACCAGGGGG
TCTGATCTGCCTGTCCCACATCAAGTGCACGCCCAAGATGAAGAAGTTCATCCCAGACGCTGCCACACCTACGA
AGCGACAAAGAGTCCGCACAGGGCGGCATAGGCGAGCGATCGTCGACATTCCTGAGATTCCTGGGTTCAAGGA
CTTGGAGCCATGGAGCAGTCATCGCACAGGTCGATCTGTGTGTGGACTGCACAACTGGCTGCCTCAAAGGGCT
TCCAACGTGCAGGTTCTGACCTGCTCAAGAAGTGGCTGCCGCAACGCTGTGCGACCTTGCCAGCAAGATCCAG
GGCCAGGTGGACAAGATCAAGGGGGCCGGTGGTGACTAAA<u>CACTCGAGTAGCGCTAGGCCTAC</u><u>**AAACACCAT**</u>
<u>**TGTCACACTCCAACAAACACCATGTCACACTCCAACAAACACCATTGTCACACTCCA**</u>
